# Mental Health Needs Assessment During the COVID-19 Pandemic: Consensus Based on Delphi Study

**DOI:** 10.3389/fpubh.2021.732539

**Published:** 2021-10-20

**Authors:** Irena Makivić, Vesna Švab, Špela Selak

**Affiliations:** Prevention and Promotion Programmes Management, National Institute of Public Health, Ljubljana, Slovenia

**Keywords:** needs assessment, mental health, health plan implementation, COVID-19, pandemic

## Abstract

The COVID-19 pandemic has revealed significant gaps in mental health in terms of unrecognized and unmet needs. The goal was to accurately assess the needs and identify gaps in this area during the epidemiological crisis. A Delphi study to identify the needs was conducted with a group of decision-makers, experts, and users of mental health services. A starting point of the Delphi study was prepared in two working groups, based on recognizable international recommendations and experiences of the practitioners from the field situation. This initial set of emergency measures was supplemented through the first Delphi round, and consensus about the importance was reached in the second round. A total of 41 activities were derived, the vast majority of which were rated with a score of 4 or more. Mental health activities, which should be addressed in terms of needs, can be divided into systemic measures and service measures. This study recognizes a need to reorganize services in the direction of improving local accessibility and strengthening the network of services for immediate responses to the psychological, health, and social needs of individuals, including those arising from crisis situations, such as COVID-19 pandemic. The results of this study are in line with the international recommendations and also influenced the formulation of the Action Plan of the National Mental Health Program, while some of the measures were already implemented during the publication of the research results.

## Introduction

The COVID-19 pandemic has affected the mental health of populations around the world. Evidence from the outbreaks of SARS, Ebola, Influenza A H1N1, and MERS has shown their impact on psychological distress (1–5), which was also recognized from the beginning of the COVID-19 pandemic (6–12). Feelings of bereavement, isolation, and fear during pandemics, as well as loss of income, increased the risk of developing insomnia, anxiety, and depressive symptoms. People at the risk of mental disorders may experience a relapse with regard to a range of mental and substance-use disorders (6, 10, 13). Pre-existing mental disorders increase the risk of becoming severely physically ill or even dying, as well as the risk of having long-term complications due to COVID-19 (14, 15). Widespread psychological distress was reported in many populations during the reported period, and a long-term increase in the number and severity of mental health problems is possible (16). The effects of the COVID-19 pandemic on mental health are as important as understanding its clinical features, transmission patterns, and the overall management of the outbreak (17).

While the COVID-19 pandemic increased the demand for mental health services, the WHO nevertheless reported that mental health services have been disrupted or halted in 93% of countries worldwide, including Slovenia. The WHO identified mental health as an integral component of COVID-19 responses, and recommended allocating more resources in order to maintain or increase mental health services during the pandemic (13). Moreover, based on the growing trend of mental health problems in many European countries, it is necessary to scale-up mental health services (18), so they can better match the actual mental health needs.

In 2018, the National Mental Health Program (NMHP) 2018–2028 was adopted in Slovenia (19) to reform the mental health provisions with the aim of achieving comprehensive, community-oriented, and needs-led services according to the international recommendations. During 2020, the National Institute of Public Health in Slovenia started to coordinate the second Action Plan to implement the NMPH from 2021 to 2023 including a crisis plan regarding the COVID-19 pandemic. Psychiatric services are currently organized in outpatient clinics, psychiatric hospitals, and in the newly developed community-based mental health centers at the primary health care level. During the crisis, improved accessibility was recognized as an essential factor in reducing the mental health burden, and a general recommendation was made to organize services in the community.

Given the increased needs caused by the pandemic, different problems with regard to accessing care can be addressed through the organization of efficient, affordable, and human rights protective community-based services as a part of any national COVID-19 recovery plan (16). Obstacles to accessing mental health services should be removed. The stigma and discrimination (20) that seemed to increase during the times of crisis should also be reduced (21). An assessment process with all stakeholders, i.e., service users and providers from different professional groups and purchasers (primarily politicians), is needed to provide evidence-based solutions for an effective consensus-based planning (22). User involvement is essential both in decision-making as well as within the implementation (23), through the involvement of peer support (24) and with addressing the needs of different vulnerable groups. Mental health services should also be scaled up because the COVID-19 pandemic produced greater social isolation which increases the risk of domestic violence and poor mental health (25). Educational programs and humanitarian assistance supporting vulnerable groups are needed. Organized support should also be accessible for overburdened frontline workers (26). The majority of the actions mentioned above are also suggested in the United Nations guidelines (16), which define a set of actions to urgently minimize the mental health consequences of the pandemic. Specifically, these call for a whole-society approach to promote, protect, and care for mental health, ensure the widespread availability of emergency mental health and psychosocial support, and support recovery from COVID-19 by building mental health services for the future.

This study aimed to identify and design a set of emergency mental health activities needed in the Slovenian mental health care system through a consensus-based process. The present study serves as an evidence-based approach to plan further actions in this context. The results are also a part of a formative evaluation process for further steps in the mental health field. Some Slovenian research also examines the health field using a Delphi study (27), as do some international studies (28–34), along with addressing the mental health of an individual (35), but none has carried out a mental health needs assessment during the pandemic. Research about mental health services in this context is also an opportunity (36) for service organization improvements and to address mental health needs with the engagement of professional organizations, a political review of mental health plans and policies, and consultation with service users (37).

## Materials and Methods

To gain the highest level of consensus among professionals working in the field of mental health, the Delphi method for gathering data from respondents focusing on their domain of expertise (38) and achieving consensus on measures was used.

Two interdisciplinary working groups, formed within the implementation of the Resolution on the Mental Health Program 2018–2028, were asked to suggest potential participants to take part in the Delphi study. The participants, invited from all key stakeholder groups, were chosen due to their reputation, skills, and role in the field of mental health. The selection of the participants was additionally supplemented by the snowball technique to prepare the final list. Therefore, we invited 128 participants from whom the majority (53.9%) were service providers, and the rest were decision-makers (25.8%) and service users (20.3%). None of the invited participants were excluded, only those who did not want to participate and did not sign the agreement were not included in the first round of the Delphi study.

**Figure 1:**
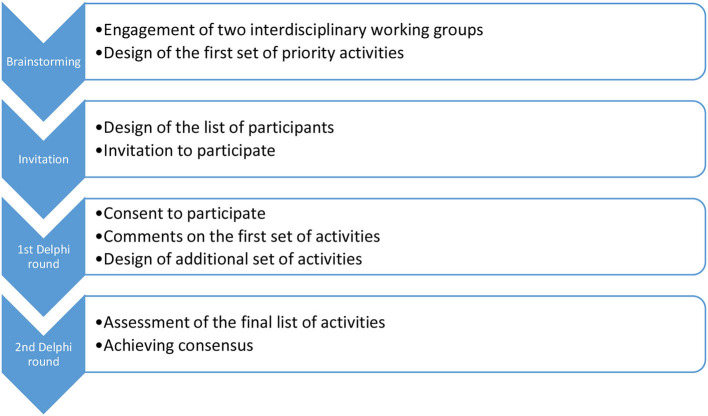
Workflow of the Delphi study.

The first set of priority activities during the COVID-19 pandemic was listed by two interdisciplinary working groups (Figure 1) gathered together to prepare starting points for mental health needs assessment. The first group addressed the network of services and the second addressed the mental health education. Together they had a total number of 30 members, including decision-makers, service providers, and service users equally represented. Those groups prepared a list of proposed activities, representing different measures in the field of systemic regulation and management of mental health. This initial list was sent to 129 participants who were invited to participate in the Delphi study, to make a review, and add additional comments. The purpose of this first round of the Delphi study was to obtain comments on the proposed activities and gather proposals for additional activities considered necessary in the system in the long run, and emergency ones that need to be implemented as soon as possible. After the first round, all comments and suggestions were reviewed and taken into consideration when designing the final set of activities, including proposals for additional measures. All commented and proposed activities were combined in terms of content and meaningfully formed into the final set of activities that was sent to the second and last rounds of the Delphi study to all participants who participated in the first round. In the second round, participants rated the importance of the listed activities on a 5-point Likert scale from 1 (not important at all) to 5 (very important). The aim of the last round of the Delphi study was to reach a consensus (at least 70% agreement) on the importance (score 4 or 5) of each activity. For the purpose of the last round, the statistical analysis was used. We calculated arithmetical means and modes for all activities that were assessed by the participants in the second round. Consensus was based on the percentage of those who assessed the activity with the grade of 4 or higher.

## Results

With regard to the first Delphi round, from all the invited participants, more women (40) than men (9) responded to the invitation and participated in the study, and the overall response rate of the first round was 37.9%. There were 11 (22.4%) representatives of policy- or decision-makers, 28 (57.2%) representatives of professionals in the field of mental health (all professions), and 10 (20.4%) representatives of service users or relatives. All those who participated in the first round were asked to assess the importance of each activity. We received 38 responses in the second round, representing a high (77.6%) response rate. There were 7 (18.4%) representatives of decision-makers or politicians, 23 (60.5%) representatives of professionals, and 8 (21.1%) representatives of service users or relatives, with a total of 33 women and 5 men participating in the second round.

The initial list contained 53 activities, representing 12 measures covering different fields of service organization supplementing the network of services and education. The list was sent to the first Delphi round. The final set was composed of 41 activities within 16 different measures. The final set was included in the last Delphi round to be assessed with regard to the level of importance.

The study highlighted the most important activities according to the recognized needs in the field of mental health during and after the COVID-19 pandemic. These are as follows: (A) Local action groups should be developed or scaled-up to achieve the delivery of food, medicines, and protective equipment to the homes of the people, as well as companionship for those who are lonely, isolated, and excluded because of poverty. The core actions are to be taken through NGO networks and social-care centers. Protocols for connected and continuous outreach are needed, especially for vulnerable groups, such as children, the elderly, and people with preexisting mental disorders. Online and telephone access should be provided to ensure connections among service providers and users. (B) Regular and mandatory consultations should be established among health, social, and educational services, among all sectors with the inclusion of humanitarian and civil society organizations to assess the needs for mental health interventions in the regions, and to enable these to be delivered locally and in good time. (C) A single entry point is necessary to access the psychological support interventions with an accessible telephone number and 24-h information, support, and crisis counseling. (D) Accessible and highly professional psychological assistance for traumatized and overstressed individuals working in the frontlines of treatment and care is needed, as well as specialized care for those infected with COVID-19. The solution chosen as the most effective was to establish educational programs and support for different intensity levels of care (from self-help and mutual support to highly qualified psychotherapy). In addition to lay training about the management of problems related to insecurity, fear, and stress, regular supervision of professionals is necessary, especially during a crisis. More specifically, continuous training and supervision are needed for professionals in health, social, and educational services, especially for those working with vulnerable groups (the lonely, elderly, children and adolescents, victims of violence, people with long-term mental health problems, and other vulnerable groups). Peer support has proven to be successful in Slovenia and is also listed among the priority actions. (E) Destigmatization campaigns need to be continuously implemented to improve help-seeking behaviors. (F) Services addressing domestic violence need to be strengthened. Safe points for reporting such violence need to be established and their visibility must be improved. (G) In times of crisis, the risk of human rights violations is high, so there is an important call to strengthen the advocacy and representation services for people with mental health problems and to monitor the protection of their rights, including institutional and hospital settings. (H) To expand the network of accommodation facilities with different levels of support for people in need, to increase the number of (smaller) group homes or housing units for people with mental disorders (especially in areas where these facilities do not exist), and to strengthen the network of day-care centers. (I) To strengthen the network of vocational rehabilitation and supported employment facilities as well as the promotion of social entrepreneurship, because of the deep economic crisis. (J) The need to provide accessible transport for a group of people with mental health problems was also listed among the priorities. (K) Protective equipment must be provided for the related field work (for health workers, social workers, including those in accommodation programs, and for relatives or carers of people with mental health problems), as well as equipment for remote access.

All the given activities were rated on average with a score of 4.3 on the 1-to-5 scale of importance, but most often with a score of 5 (mode of all overall scores). On average, the percentage of agreement or consensus level was 84.3% (from 71.1 to 97.4%). In the vast majority of cases, all participants evaluated all the statements, and only one person did not give a rating for only three statements. On average, all but one of the activities were rated 4 or higher. Only one activity, addressing the work of mental health councils, had an average score of 3.9. Participants, on average, expressed the opinion that all activities are important or very important. Given the high level of consensus reached, the third round was not necessary.

## Discussion

**Figure 2:**
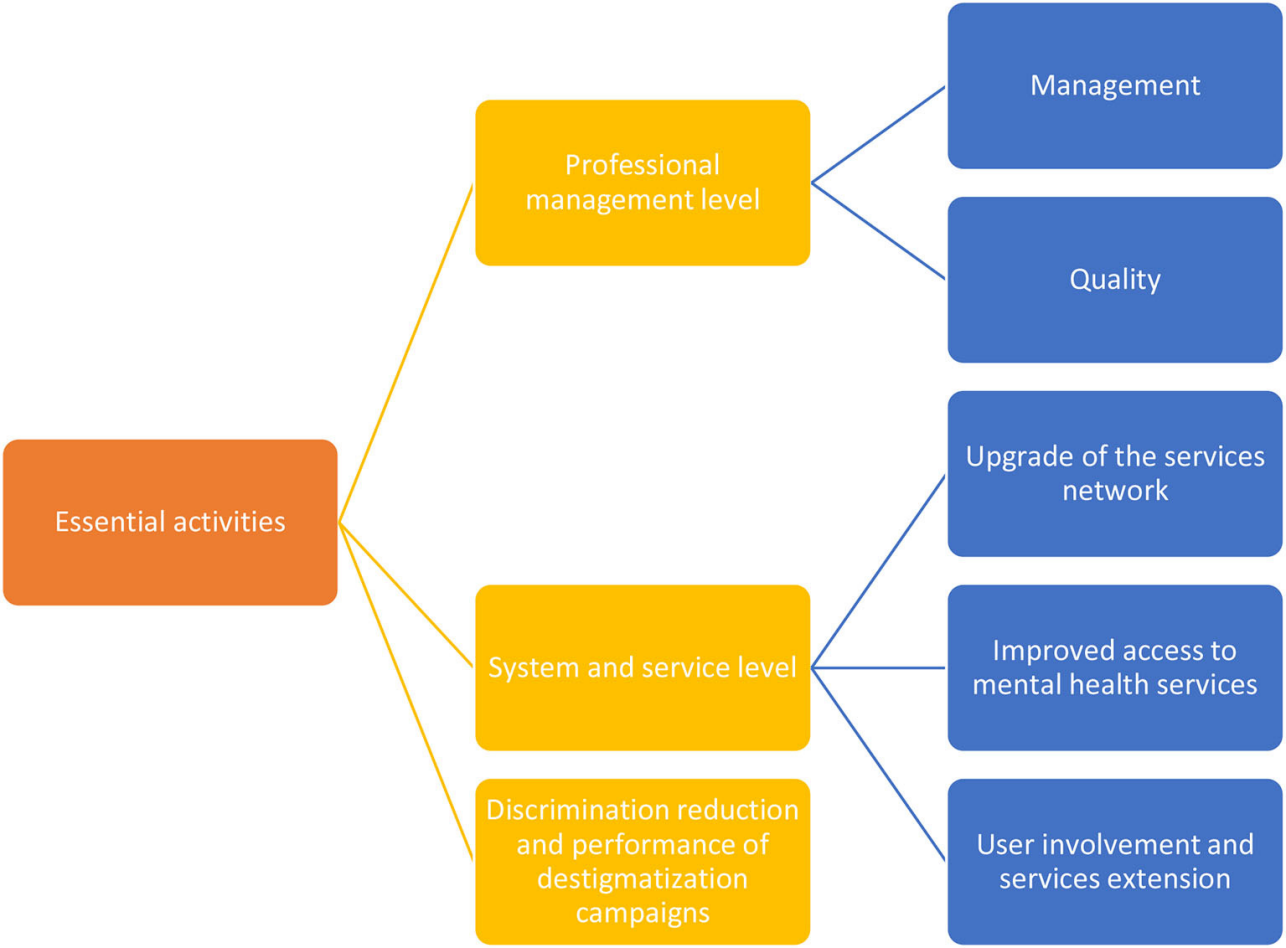
Essential activities during and after the COVID-19 pandemic.

The article describes the Delphi study conducted to identify the needs and gaps in mental health service delivery during the period of the COVID-19 pandemic in Slovenia. The study identified various systemic and service measures that need to be addressed and/or strengthened. Reports of mental health interventions during the COVID-19 pandemic are multiplying rapidly. Several studies (39–43) have so far suggested different activities, measures, and recommendations to address mental health challenges during the pandemic, but to the best of our knowledge, none has used a scientifically supported method, such as the Delphi technique. The results of our Delphi study are in line with the international guidelines (44) using the existing knowledge about the problem from reports and scientific databases (45), whereas in our study, the Delphi-based consensus was used, i.e., an open and thorough discussion with stakeholders to highlight the essential activities according to the recognized needs in mental health during and after the COVID-19 pandemic, and to identify various systemic and service measures that need to be addressed. Based on the results, the recognized needs in mental health during and after the COVID-19 pandemic are as follows (Figure 2): (1) Professional level: better management (coordinated work of mental health councils and local health action groups for accessible, needs-based support) and quality (addressing highly professional support through adequate educational programs and supervisions). (2) System and service level: upgrade of the services network (mental health centers organized locally, accommodation facilities, vocational rehabilitation, and day centers), improved access to mental health services (coordinated protocols for different stakeholders, transportation, and protective equipment within the COVID-19 pandemic and a single entry point for people with mental health problems), user involvement, and service extension (lay assistance and promoting peer support advocacy as well as addressing the risk of human rights violations bearing in mind all the vulnerable groups, improvement of services working against domestic violence and other forms of abuse). (3) Reducing discrimination and promoting destigmatization.

The COVID-19 pandemic revealed many gaps in service provision and put a new emphasis on the community mental health care model (46). A locally accessible service network was seen as one of the highest priorities in this context, together with local and central government and the coordination of services, including protocols for communication among different services. The need to address the needs of the people at their homes was also an important priority because of the obviously inadequate protection of people with mental health disorders in institutions (47). The consensus reached by the Delphi study is in line with this recognition, and is as such, another important call for mental health reform in Slovenia. The need for social protection of service users is included in the Delphi results, including the terms of accommodation, provision of life necessities, and vocational rehabilitation, transport, and employment, as stressed in the last WHO recommendations about community care (47). Human rights protection with adequate direct involvement of service users in the planning and delivery of services was once again stressed, but with little reflection in the subsequent political decisions (48). Moreover, before the Delphi study in Slovenia, mental health experts from different backgrounds along with volunteers provided a single entry point which was established as a national helpline (49), which is supported and complemented by the local mental health psychological support offered within health promotion centers (50). Furthermore, information and support materials, including guidelines, were developed for professionals and the general public on a large-scale basis, addressing different aspects of mental health promotion and prevention during the pandemic, some also in the languages of the various national communities in Slovenia (51).

The major strength of this study is the inclusion of policy-makers, service users, and mental health professionals. The Delphi results call for reforming mental health services in line with international recommendations (52, 53) and measures abroad, and we find our results comparable to those reported in other countries, as well as applicable to our context. The Delphi consultation achieved the participation of service users, professionals, and providers in the codesign of emergency measures. Particular attention should be focused on additional services, especially for vulnerable groups. The findings are important to address the knowledge gap in the field of mental health needs during pandemics. The results of the current research call for good collaboration among the existing services and sectors, as well as a shift toward a community-based approach. However, despite the rigorous methodology that was used, the present research has some limitations, as follows. Namely, the Delphi study did not include health- and social-care professionals working in the frontlines with COVID-19 patients at the beginning of the pandemic. It is possible that we addressed more individuals who are already engaged in this field but do not necessarily know all the disadvantages of the current system that should be addressed. We also do not know if we included a large group of people with mental health needs to address their different views on the focal problems in a sufficiently comprehensive manner. Nevertheless, we repeatedly searched for representatives of all stakeholder groups and tried to obtain a more balanced group of participants. Another limitation is that the primary-care teams working during the pandemic were not specifically addressed, with regard to the training and guidelines in the mental health field. With the increase in telemedicine services across the globe, there is also a need to address the problems that can arise with these. In the discussion part of this study, the need for better online and telephone access to improve the connections between services and users were mentioned, but the limitations and challenges that can occur with this were not addressed. There is likely to be a need for new guidelines that can help manage this new context for the provision of services.

## Conclusions

Our main finding is that a consensus about emergency mental health activities during a pandemic or other natural disaster is needed, and this can be constructed with the Delphi method. The actions already launched to meet the mental health needs of the population in Slovenia during the COVID-19 pandemic correspond well with the results of this study. Nevertheless, continuous consultation among experts, politicians, and service users is needed to make the right decisions at the right time, according to our experience. If the Delphi recommendations were strictly followed in Slovenia, and followed in a timely manner, we could overcome many challenges.

## Data Availability Statement

The raw data supporting the conclusions of this article will be made available by the authors, without undue reservation.

## Ethics Statement

Ethical review and approval was not required for the study on human participants in accordance with the local legislation and institutional requirements. The patients/participants provided their written informed consent to participate in this study.

## Author Contributions

IM lead methodology and carried out field work. IM and VŠ recoded answers and make analysis. ŠS wrote introduction. IM wrote methodology section. VŠ wrote conclusion. All authors contributed to the article and approved the submitted version.

## Funding

This work was supported by the Slovenian Research Agency, Project No.: Z3-2652.

## Conflict of Interest

The authors declare that the research was conducted in the absence of any commercial or financial relationships that could be construed as a potential conflict of interest.

## Publisher's Note

All claims expressed in this article are solely those of the authors and do not necessarily represent those of their affiliated organizations, or those of the publisher, the editors and the reviewers. Any product that may be evaluated in this article, or claim that may be made by its manufacturer, is not guaranteed or endorsed by the publisher.
